# The evaluation of intracellular energy metabolism in prediabetic patients and patients newly diagnosed with type 2 diabetes mellitus

**DOI:** 10.3906/sag-1912-60

**Published:** 2021-02-26

**Authors:** Erkan DULKADİROĞLU, Hüseyin ÖZDEN, Hüseyin DEMİRCİ

**Affiliations:** 1 Department of Internal Medicine, Kırşehir Training and Research Hospital, Kırşehir Turkey; 2 Department of General Surgery, Faculty of Medicine, Ahi Evran University, Kırşehir Turkey; 3 Department of Endocrinology, Medicana International Hospital, Ankara Turkey

**Keywords:** Prediabetes, type 2 diabetes mellitus, intracellular energy, leukocyte

## Abstract

**Background/aim:**

Increased susceptibility to infections is a serious problem in diabetics. Impairment in the energy metabolism of the immune system is the main source of the problem. Early diagnosis of the impairment in energy metabolism is crucial. Our study aimed to investigate the energy metabolism in leukocytes in patient groups such as prediabetics and patients newly diagnosed with type 2 diabetes mellitus.

**Materials and methods:**

Our study included 21 newly diagnosed type 2 diabetic patients (NDDP), 30 prediabetic patients, and 22 adult volunteers. 75 g oral glucose tolerance test (OGTT) was applied to all patients included in the study. Blood samples were taken after 9-16 h of fasting and fasting blood glucose (FBG), postprandial blood glucose (PBG) levels, total cholesterol (TC), triglyceride (TG), high-density lipoprotein (HDL), fasting serum insulin, and hemoglobin A1c (HbA1c) levels were evaluated. After the cells were completely lysed, citrate levels from the released mononuclear leukocyte cells (MNC) content were manually studied, and lactate levels were applied to the autoanalyzer with the lactate kit. Lactate and citrate results were calculated as µg/mL. Statistical comparisons were done using Chi-square test, Mann-Whitney U test and student’s t test, and P < 0.05 values were accepted as significant.

**Results:**

A significant difference was found between the controls and the other groups (newly diagnosed diabetic patients (NDDP), impaired fasting glucose (IFG), and impaired glucose tolerance (IGT)) in terms of FBG levels (P < 0.001, P < 0.001 and P < 0.001, respectively). IFG and IGT patients had significantly higher PBG levels compared to the control group (P = 0.009 and P < 0.001, respectively). There was a significant difference between the IFG and IGT patients in terms of insulin levels (P = 0.019). There was a significant relationship between FBG levels and lactate production only in the NDDP group (r = 0.610, P = 0.003)

**Conclusion:**

The metabolic effects of hyperglycemia on leukocytes is in direction of anaerobic glycolysis. The increased anaerobic pathway is closely related to blood glucose levels and insulin resistance.

## 1. Introduction

Diabetes is a chronic metabolic disease where the organism cannot make sufficient use of carbohydrates, fats, and proteins due to insulin deficiency or defects in insulin effectiveness requiring constant medical care [1]. Increased susceptibility to infections is a challenging problem in diabetic patients. It has been shown that a variety of mechanisms of innate immune system are impaired in these patients. Lactate production is considerably decreased in the neutrophils of diabetics, whereas cytosolic calcium levels increase in neutrophils. Chronic and persistent calcium entry into the cell occurs and leads to inhibition of mitochondrial oxidation and reduction in the amount of ATP [2-5]. Deterioration in the energy metabolism of immune system plays a crucial role in the development of infections [6].

Glucose can be used in glycolysis or oxidative phosphorylation for the production of adenosine triphosphate (ATP). Glycolysis occurs in the cytosol, and 2 molecules of pyruvate are formed from 1 molecule of glucose. 2 ATP is obtained from each molecule of glucose and is oxygen independent. Pyruvate turns into lactate. Alternatively, oxidative phosphorylation is oxygen dependent and occurs in mitochondria. It consists of two reactions: conversion of intermediate molecules (pyruvate and fatty acid) to acetyl coenzyme A (Acetyl CoA) and reduction of acetyl CoA to carbon dioxide (CO2) in the tricarboxylic acid cycle. Net 30-32 ATP is earned for each glucose [7]. Citrate is a marker used as an indicator of oxidative mechanism. In this case, oxidative phosphorylation seems to be more efficient in ATP production. Diabetic patients are known to be susceptible to infections. One of the reasons for this increased susceptibility to infections may be a decrease in the oxidative mechanism, a decrease in the nonoxidative mechanism, or a decrease in both mechanisms compared to healthy volunteers [6].

The early diagnosis of the impairment in intracellular energy metabolism in diabetic patients is extremely important. Our study aimed to evaluate the levels of aerobic and anaerobic oxidation products in leukocytes of adult prediabetic and newly diagnosed type II diabetes mellitus patients. The relationship between the severity of diabetes and intracellular energy metabolism was also investigated in our study. 

## 2. Materials and methods

Written approval was obtained from the Local Ethics Committee of the Faculty of Medicine in Kırıkkale University on 28th April 2011 with the number 2011/0056. Our study consisted of 21 newly diagnosed adult type II diabetes patients and 30 prediabetic patients aged 19–70 years. Patients who applied to the endocrinology policlinic of the Kırıkkale University Medical Faculty Hospital during May 2011 were included in the study. None of our patients were receiving diabetes control treatment. The control group consisted of 22 adult volunteers who were not morbidly obese or diabetic, and they were with no additional disease and drug use. Volunteers who were younger than 18 or older than 70 years, who were previously diagnosed and treated diabetics, morbidly obese, who had cardiovascular and cerebrovascular diseases or organ insufficiency such as chronic renal insufficiency, respiratory insufficiency and heart insufficiency, and who received chemotherapy were excluded from the study. 

 Twenty-two volunteers (16 females and 6 males) were included in the patients admitted to the outpatient clinic. 75 g oral glucose tolerance test (OGTT) was applied to all patients admitted to the outpatient clinic. The patients were divided into 3 groups as healthy individuals, prediabetics, and newly diagnosed type 2 diabetes mellitus patients according to the OGTT results. Patients were grouped as prediabetes (IFG: those with fasting plasma glucose between 100-125 mg/dL and IGT: those with second hour plasma glucose between 140-199 mg/dL in OGTT), newly diagnosed diabetes (FBG = 126 mg/dL and above and/or those with second hour blood glucose=200 mg/dL and above in OGTT) and healthy volunteers according to the results of fasting blood glucose (FBG) and OGTT 2nd h blood glucose. Blood samples were taken after 9-16 h of fasting; FBG levels, total cholesterol (TC), triglyceride (TG), high-density lipoprotein (HDL), fasting serum insulin, and hemoglobin A1c (HbA1c) levels were measured. The Olympus AU 400 auto-analyzer (Olympus Corporation, Tokyo, Japan) was used with Olympus kits. LDL level was measured using the Friedewal dequation [LDL = TK - (VLDL + HDL); VLDL = TG / 5]. The HOMA-IR calculation was used to determine insulin resistance. The homeostasis model assessment (HOMA) insulin resistance index (HOMA-IR) was calculated according to the following formula: FBG (mmol / l) X fasting insulin (μIU / mL) / 22.5.

The control samples of the study were taken from 22 volunteers who had no disease and fulfilled our control group criteria. Samples were collected as approximately 7-8 mL venous blood into 9 mL BD Vacutainer tubes with acid citrate dextrose (ACD). When the minimum target number was reached, leukocyte samples were separated from peripheral venous blood taken into ACD tubes by Ficoll method; mononuclear leukocytes were isolated, and fetal heart serum (fetal bovine serum) was added to all samples equally and frozen at -80 ºC to maintain cell viability. 

The control group was prepared with the same protocol, and Type 2 DM and prediabetes mononuclear leukocyte cells (MNC) were thawed on the scheduled date of the study and then washed with phosphate buffered saline (PBS). MNC samples were homogenized in SONICS VC-505 ultrasonic homogenizer (Sonics & Materials, Inc. USA) at 100 hertz for 5 min, and then the intended parameters were studied.

After the cells were completely lysed, citrate levels from the released MNC content were manually studied, and lactate levels were applied to the RANDOX Lactate Kit Olympus AU 400 autoanalyzer (Olympus Corporation). Lactate and citrate results were calculated as µg/mL. Researchers had no information about which group the samples belonged to until the concrete data was obtained from the study.

At the beginning of the study, it was planned to take the samples into fluoride tubes for the inhibition of glycolytic enzymes from the time of collection, to keep the current substrate levels of the cells constant and to ensure the necessary enzyme inhibition (with HCl) until the measurement time in leukocyte suspension after leukocyte separation. The rate of metabolism was evaluated using lactate levels for anaerobic glycolysis and citrate levels for aerobic glycolysis. 

After the formation of pentabromo acetoneas result of oxidation and bromination of citric acid, modified Cahors method based on spectrophotometric measurement was used for the measurement of Citrate levels in mononuclear leukocyte.

### 2.1. Statistical analysis

SPSS 15.0 software for Windows (SPSSInc., Chicago, IL, USA) was used for statistical analysis. Frequency Tables are presented for categorical variables, and descriptive statistics (mean, std. Deviation, minimum, maximum) are presented for numerical variables. The comparison of continouos variables among four groups were done with Kruskall-Wallis test and the binary comparisons were done with Mann-Whitney u test with Bonferroni correction. In binary comparisons, according to Bonferroni correction, P value <0.008 accepted as statistically significant. The categorical variables were compared with Chi-square test. The correlation analysis was done with spearman correlation test.

## 3. Results

There were 21 newly diagnosed diabetic patients (NDDP), 7 with impaired fasting glucose (IFG), 23 with impaired glucose tolerance (IGT), and 22 healthy control volunteers (CP). The study groups consisted of 44 (60.3%) females and 29 (39.7%) males. There was no significant difference between the groups in terms of sex. The groups were similar in terms of sex, serum LDL, HDL, cholesterol, and body fat percentage (BFP), detailed in Table 1.

**Table 1:** Mean values of demographic characteristics of the study groups.

Mean values	Control	NDDP	IFG	IGT	P
AGE (year)	41.95 ± 7.96	50.48 ± 8.75	47.39 ± 11.24	42.29 ± 10.04	0.03 a 0.128 b 0.798 c
LDL mg/dL	120.45 ± 43.77	130 ± 34.48	122.22 ± 32.65	110.14 ± 35.06	0.444 a 0.820 b 0.628 c
HDL mg/dL	49.68 ± 8.05	44.71 ± 10.79	49.87 ± 10.81	46 ± 10.93	0.145 a 0.820 b 0.230 c
Cholesterol mg/dL	196.18 ± 50.02	223.9 ± 35.95	198.26 ± 38.93	207.14 ± 32.66	0.078 a 0.937 b 0.308 c
Triglyceride mg/dL	129.64 ± 73.79	244 ± 183.68	143.96 ± 63.36	237.57 ± 114.13	0.001 a 0.192 b 0.008 c
BFC	29.7 ± 9.59	32.75 ± 8.73	34.32 ± 9.78	28.67 ± 12.82	0.325 a 0.089 b 0.980 c
BMI	26.10 ± 4.08	30.51 ± 5.06	29.72 ± 4.22	31.22 ± 3.67	0.004 a 0.007 b 0.008 c

BFC: body fat percentage. BMI: body mass ındex.

The mean age was significantly higher in the NDDP group compared to healthy controls. However, all other groups were statistically similar in terms of mean age. Among all groups, the highest triglyceride level was found in the NDDP group. There was a statistically significant difference between the NDDP group and the IFG and CP groups in terms of triglyceride levels. The highest total cholesterol level was found in the NDDP group. There was no statistically significant difference between the groups in terms of total cholesterol. The lowest HDL level was noted in NDDP group, and the highest HDL level was noted in the healthy control group. There was no significant difference between the groups in terms of HDL levels. The highest LDL level was noted in NDDP group and the lowest LDL level was noted in IGT group. There was no statistically significant difference between the groups in terms of LDL levels. Body mass index (BMI) was the highest in IGT group. There was a significant difference between the controls and the NDDP, IFG, and IGT groups in terms of BMI, detailed in Table 1.

Mean fasting blood glucose (FBG) levels were noted as 175.57 ± 62.72 mg/dL in the NDDP group, 106.57 ± 5.14 mg/dL in the IFG group, 100.14 ± 12.41 mg/dL in the IGT group and 89.32 ± 6.96 in the control group. There was a significant difference between the controls and the NDDP, IFG and IGT groups in terms of FBG levels (P < 0.001, P < 0.001 and P < 0.001, respectively). FBG levels were found to be significantly lower in controls compared to the other groups. Mean FBG levels were found to be significantly higher in the NDDP group.

Postprandial blood glucose (PBG) levels were noted as 218.71 ± 84.42 mg/dL in NDDP patients, 151.53 ± 7.78 mg/dL in IGT patients, 102.83 ± 0.82 mg/dL in IFG patients, and 85 ± 21.41 mg/dL in control patients. There was a significant difference between the groups in terms of PBG levels. Mean PBG levels were found to be significantly higher in the NDDP group (P < 0.001). IGT patients had significantly higher PBG levels compared to IFG patients (P < 0.001). IFG and IGT patients had significantly higher PBG levels compared to the control group (P = 0.009 and P < 0.001, respectively).

Mean HbA1c levels were noted as 8.18% ± 2.48% in NDDP patients, 5.91% ± 0.5% in IGT patients, 5.91% ± 0.34% in IFG patients and 5.80% ± 0.32% in control patients. Compared to the other groups, NDDP patients had significantly higher HbA1c levels (P < 0.001). There was no significant difference between the other groups (IFG, IGT and CP) in terms of mean HbA1C levels. 

Mean insulin levels were measured as 19.21 ± 14.7 mIU/mL in the NDDP group, 17.95 ± 7.80 mIU/mL in the IGT group, 10.82 ± 4.45 mIU/mL in the IFG group and 11.87 ± 5.04 mIU/mL in the control group. There was only a significant difference between the IFG and IGT groups. 

The insulin resistance characteristics of the study groups were evaluated. Insulin resistance levels were found to be the highest (85.7%) in the NDDP group. It was 71.4% in IGT patients, 52.2% in IFG patients and 40.9% in control patients. Insulin resistance levels of NDDP patients were found to be significantly higher compared to IFG and control patients (P = 0.017 and P = 0.002, respectively).

When the parameters examined for all groups are evaluated. There was a significant correlation between HbA1c and lactate levels (r = 0.246, P = 0.036) but there was no significant correlation between HbA1c and citrate levels (r = -0,121, P = 0,315). There was a significant correlation between FPG and lactate levels (r = 0.245, P = 0.037) but there was no significant correlation between FPG and citrate levels. (r = -0.122, P = 0.302). There was no significant correlation between PPG, lactate (r = 0.179, P = 0.129) and citrate levels (r = -0.083, P = 0.483). There was no significant correlation between insulin, lactate (r = -0.152, P = 0.199) and citrate levels (r = -0.028, P = 0.813). There was no significant correlation between insulin resistance, lactate (r = -0.103, P = 0.388), and citrate levels (r = -0.076, P = 0.520). Comparisons of lactate and citrate levels were detailed in Table 2.

**Table 2:** Comparisons of the mean lactate and citrate levels of the groups.

	Control	NDDP	IFG	IGT	P
Lactate µg/mL	0.212 ± 0.147	0.236 ± 0.204	0.246 ± 0.215	0.214 ± 0.206	0.950
Citrate µg/mL	82.28 ± 20.09	78.41 ± 15.60	79.53 ± 18.41	79.23 ± 16.56	0.974

NDDP: newly diagnosed diabetes patients; IFG: impaired fasting glucose, IGT: ımpaired glucose tolerance.

**Figure 1:**
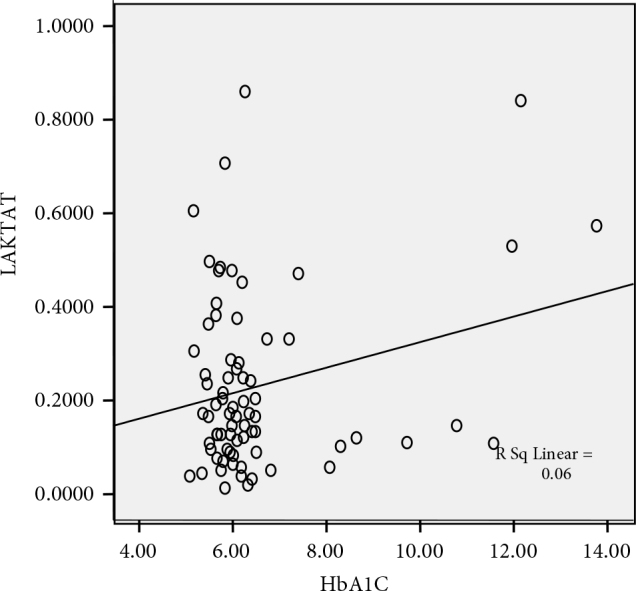
Relationship between the HbA1 levels and lactate production.

**Figure 2:**
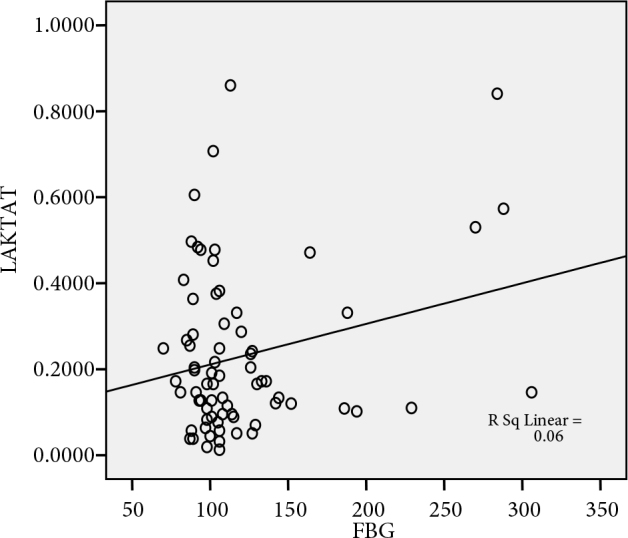
The relationship between the FBG levels and lactate production in NDDP group.

Our study showed that there was a positive significant correlation between HbA1c and FBG levels (r = 0.902, P < 0.001), postprandial BG levels (r = 0.841, P < 0.001), lactate levels (r = 0.246, P = 0.036), (Figure 1), insulin resistance (r = 0,243, P = 0.039), serum cholesterol levels (r = 0.264, P = 0.024) and triglyceride levels (r = 0.324, P = 0.005). However, when the groups were examined separately, there was a statistically significant positive correlation between HbA1c levels and FBG levels, postprandial BG levels, and lactate production in the NDDP group. There was no correlation between HbA1c and citrate production. When all groups were examined separately, the correlation between FBG levels and lactate production increased only in the NDDP group (r = 0.610, P = 0.003), (Figure 2).

## 4. Discussion

The end product of glycolysis is pyruvate in cells with mitochondria and enough oxygen. In the absence of sufficient oxygen, glucose turns into lactate. Pyruvate, the final product of pyruvate dehydrogenase glycolysis, converts irreversibly into acetylCoA, the basic fuel of citric acid cycle and the building block of fatty acid synthesis [8]. Lactic acidosis is caused by a number of reasons, including pyruvate dehydrogenase deficiency [9]. Pyruvate dehydrogenase activity in the lymphocytes of patients with type 2 diabetes mellitus (type 2 DM) is less compared to healthy patients [10, 11]. It can be used in glucose, glycolysis or oxidative phosphorylation for the production of adenosine triphosphate (ATP) [12]. Glycolysis occurs in cytosoland 2 molecules of pyruvate are formed from 1 molecule of glucose. 2 ATPs are obtained from each molecule of glucose and oxygen is independent. The pyruvate is converted to lactate and nicotinamide adenine dinucleotide (NADH) is re-oxidized to form nicotinamide adenine dinucleotide (NAD). Alternatively, oxidative phosphorylation is oxygen dependent and occurs in mitochondria. It consists of two reactions: transformation of intermediate molecules (pyruvate and fatty acid) to Acetyl Coenzyme A (Acetyl CoA) and reduction of acetyl CoA to carbondioxide (CO2) in the tricarboxylic acid cycle. Free electrons are carried by NADH and the reduced flavin adenine dinucleotide (FADH2). Electrons are transferred to the electron transfer chain. It results in the protons coming out of the mitochondrial matrix. This electrochemical potential is used in the production of ATP with ATP synthase, and a net 30-32 ATP is gained for each glucose. In this case, oxidative phosphorylation is seen to be more efficient in ATP production. In this study, we investigated the shift of the energy metabolism in the neutrophils of diabetic patients to the aerobic or anaerobic direction.

Studies focused on the immune response in diabetics first began to be reported in the 1960s. Then, it was shown that impaired PMNL chemotaxis during ketoacidosis reached normal levels as ketoacidosis improved. In the early 1970s, Mowat and Baum showed that in in vitro conditions, PMNL chemotaxis index was low in diabetic patients [13]. Molenaar et al. showed that PMNL chemotaxis index was impaired in the first-degree relatives of diabetic patients [14]. Alexiewicz et al. reported that the ability of phagocytosis in PMNL obtained from newly diagnosed type 2 diabetics decreased, which was related to glycemic control, and that glycemic control obtained by a 3-month treatment also improved phagocytosis functions [15].There are many disorders in the immune system of diabetics; however, the most important factor causing susceptibility to infections is the deterioration in leukocyte functions [16].

It has been shown that various aspects of inflammation are impaired in the process of diabetes [17]. Martin et al. and Esman found a decrease in lactate production in the neutrophils of diabetic cases [2,18]. In addition, lactate production was found to be reduced by 24% when compared to controls in the neutrophils of diabetic rats [3]. In this study, we investigated the energy metabolism of mononuclear leukocyte cells in prediabetics and patients with Type 2 DM. When the groups were compared with each other, no difference was found between citrate and lactate values, indicating that there was no difference in terms of the use of aerobic and anaerobic pathways in leukocytes.

A statistically significant correlation was found between HbA1c and FBG levels, postprandial BG levels, lactate production, insulin resistance, serum cholesterol, and triglyceride levels. However, when the groups were analyzed separately, there was a statistically significant positive correlation between HbA1c levels and FBG, postprandial BG levels and lactate production only in the NDDP group. However, there was no correlation between HbA1c levels and citrate production. These findings suggest that insulin resistance, FBG levels, postprandial BG levels, and HbA1c values are more closely related to metabolic status rather than the current diagnosis of the patient. Insulin resistance, FBG, postprandial BG levels, and HbA1c levels lead to an increase in anaerobic respiration rather than aerobic respiration. It is true that diabetic leukocytes use aerobic glycolysis, and this is also valid for normal leukocytes. When compared with normal leukocytes, the similarity in aerobic and anaerobic metabolism of diabetic leukocytes does not indicate that the energy metabolism is normal. Anaerobic lactate production of diabetic leukocytes increases in the presence of conditions such as increased insulin resistance, elevated FBG levels, increased cholesterol levels, and high HbA1C levels, indicating that diabetes becomes worse. 

As our study does not include comparisons that show the effect of environment variables, we can only estimate the effect of storage conditions on the results. Nevertheless, we can say that the stored samples were under anaerobic conditions. The fetal bovine serum added to all samples provides sufficient substrate for leukocyte cells.

The difference between the groups in terms of lactate levels, widely accepted as an indicator of anaerobic pathway, has been reported to be different in similar studies. Martin et al. and Esman found a decrease in lactate production in the neutrophils of diabetic cases [2,18].

In addition, lactate production was reduced by 24% when compared to controls in the neutrophils of diabetic rats [3]. Şahin et al. found lactate production to be significantly higher in IGT and DM groups compared to the leukocytes of healthy volunteers [6]. However, in our study, lactate production was similar among groups, whereas lactate production increased in diabetics.

There are many articles in the literature tackling anaerobic and aerobic glycolysis in leukocytes. However, it is very difficult to evaluate them together due to differences in the procedures in these studies. Glycolytic pathway enzymes were compared to determine metabolic differences, and effective results could not be reached by different researchers, since the studies did not continue in the same direction. Numerous tissue studies on glycolysis have focused on different reactions in glycolysis but there is no answer to the question of whether these rates are constant values in the same disease groups as metabolic indicators.

ATP-ADP ratios are also studied parameters to obtain a constant value. The variability of the multifactors (appropriate pH, thermodynamic state, low apoenzyme concentration, activator-coenzyme deficiency, etc.) that determine the effect of enzymes in the glycolytic pathway in different tissues rendered these classifications ineffective.

Providing the Michael constants (substrate, coenzyme, optimal pH, etc.) to the same degree made it possible to compare different samples, and the determination of glycolytic ratios by kinetic enzyme studies may determine whether the hypotheses put forward are correct. We used the substrate instead of the enzyme for the same purpose.

Our disadvantage was that the same samples could not be studied under different influences. We used all viable inanimate cells when comparing substrate levels. The metabolism of cells was slowed/stopped until study time.

Lactate and citrate levels were compared with those of the controls only once. Therefore, we could not comment on the amount of change. The absence of difference compared to healthy cells indicates that there was no difference between aerobic and anaerobic pathways. Lymphomononuclear (LMN) cells alter energy metabolism in the hyperglycemic state and shift to the anaerobic glycolysis pathway. The changing glycolytic pathway may affect the response of these cells to the immune stimulus [19-22]. It has been previously reported that LMN cells cannot effectively oxidize glucose in diabetic conditions, lymphocytes obtained from diabetic rats show decreased hexokinase and citrate synthase activity [23]. 

It has been previously shown that lactate production was significantly higher in DM and IGT groups compared to healthy controls. There was no significant difference between DM and IGT groups in our study. It is thought that the prediabetic stage may alter the cell energy metabolism. It has also been reported that the oxidation ability of lymphocytes is impaired due to high glucose levels [6].

In our study, a positive correlation was found between high blood sugar and lactate production. Similar results were reported in a study of streptozotocin-induced diabetic rat thymus lymphocytes [24]. It has been reported that lymphocytes and neutrophils use anaerobic glycolytic pathways in experimental diabetic models. Aerobic glycolysis increases after insulin treatment [3].

In this study, we tried to determine the effect of high glucose concentrations on leukocyte functions. The presence of insulin receptors in monocytes has previously been shown [25]. Insulin resistance may have a direct role in decreased LMN cell functions, and increased lactate may also play a role in insulin resistance and the development of diabetes [26].The relationship between lactate production and blood glucose levels in our study suggests a similar situation. Although there was no significant difference between lactate and citrate levels between the groups in our study, the correlation between blood sugar and lactate levels was striking, and this shows the importance of blood sugar control. The Diabetes Control and Complications Trial (DCCT) and the UK Prospective Diabetes Study (UKPDS) indicate that the same effort should be made for glycemic control in DM and IGT cases [27-29]. In the present study, there was no significant difference in the energy metabolism of diabetic, prediabetic, and normal leukocytes but the anaerobic glycolytic pathway correlated with glucose levels. This shows the importance of blood sugar regulation to prevent infection. Currently, studies on glucose metabolism, which play a key role in infections in diabetic patients, remains important. Glucose metabolism in all cells is the common pathway used to maintain cell continuity. The difference between diabetic leukocytes and normal leukocytes is the reason of the increased use of anaerobic glycolytic pathway in parallel with increased blood glucose. 

Our study has the following limitations. Firstly, our NDDP group is older than the remaining groups. It is known that aging is a main influencer on immune system function. Secondly, insulin resistance rate of control group is 40.9%.

In conclusion, this study is one of the rare studies investigating the metabolic effects of hyperglycemia on leukocytes. LMN cells follow similar metabolic pathways in diabetic, prediabetic, and control groups, the difference being the increased anaerobic pathway, which positively correlates with blood glucose levels and insulin resistance.

This adaptation mechanism begins before the emergence of diabetes. Further studies on this subject with larger groups are required to enlighten the mechanisms affecting the relation between diabetes and immune response.

In addition, a wide age range was determined in our study. It is known that with increasing age, his immunity decreases. New studies with a narrower age range are needed to support our findings.

## Informed consent

Written approval was obtained from the Local Ethics Committee of the Faculty of Medicine in Kırıkkale University on 28th April 2011 with the number 2011/0056. Certificate of approval was obtained from each patient.
